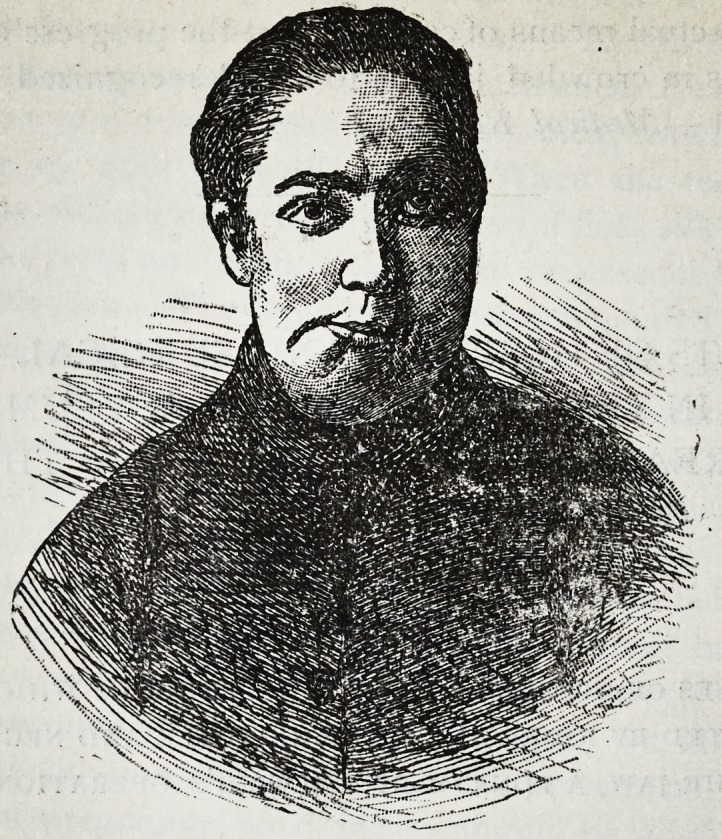# Reports of Hospital and Surgical Practice in the Hospitals and Asylums of Great Britain, Ireland, and the Colonies.—University College Hospital—Four Cases of Closure of the Jaws, and Treatment

**Published:** 1885-01

**Authors:** Christopher Heath


ARTICLE V.

### REPORTS OF HOSPITAL AND SURGICAL PRACTICE IN THE HOSPITALS AND ASYLUMS OF GREAT BRITAIN, IRELAND, AND THE COLONIES.—UNIVERSITY COLLEGE HOSPITAL.

### FOUR CASES OF CLOSURE OF THE JAWS, THREE OF WHICH WERE TREATED BY REMOVAL OF THE CONDYLE AND NECK OF THE JAW, A FOURTH BY ESMARCH'S OPERATION.

(Under the care of MR. CHRISTOPHER HEATH.)

[From notes by Mr. POLLARD, Surgical Registrar.]

CASE I. *Closure of Jaws, due to Enlargement, probably from Rheumatoid Arthritis, of the Left Neck and Condyle of the Lower Jaw: Excision of the Upper End of Ramus: Great Improvement.*—E. B., aged 36, female, was admitted on February 26th, 1883. She had had small-pox about twenty years earlier, and had occasionally suffered from chronic rheumatism in the elbows. In 1872 she had an attack of partial hemiplegia on the left side, and she then noticed that her face, which had previously been symmetrical, was drawn to the right side, and that she had difficulty in opening her mouth; the displacement of the jaw to the right side had been increasing ever 1872. She had gradually recovered the use of her limbs, but not entirely that of the left side of her face.

Her state, on admission, was as follows. The lower jaw was displaced to the right, as shown in the illustration, so that the middle of the symphysis lay vertically beneath the centre of the right pupil when the eyes were directed forwards. The symphysis was unaltered in shape. The right angle of the jaw was only half an inch below the lobule of the ear, and the continuation backwards of a line along the lower borderof the jaw passed over the tip of the mastoid process. The right condyle was apparently buried beneath the zygomatic arch, and, in place of the normal elevation, there was a depression opposite the articulation. Betwcen the left angle of the jaw and the upper border of the zygomatic arch there was a hard bony mass continuous with the jaw, and extending forwards to the molar bone, with the lower border of which it merged. The whole of the outer surface of this enlarged ramus was smooth, and over it the masseter muscle appeared to be tightly stretched and united to it by firm tissue.

The patience experienced abnormal sensations in the skin of the whole left side of the head and neck, where also tactile sensibility was diminished ; there was partial paralysis on the left side of the face, but the limbs of that side were as strong as those of the other.

March 14th. The patient being under the influence of chloroform. Mr. Heath tried to move the jaw, but, failing to make any material alteration, he made a vertical incision along the upper two-thirds of the posterior border of the ascending ramus of the jaw, and extending down to the bone, He then stripped off the soft parts from the front and back of the bone, and divided the latter with an Adam's saw. The soft parts were next cleared from the upper fragment, which was, with some difficulty, leveled out of its position with an elevator, bringing away with it a small portion of the temporal muscle ; the wound was mopped out with a solution of chloride of zinc, a plug of lint, soaked in the same, was left in it, and a dressing of iodoform and salicylic wool was applied outside. The portion of bone removed consisted of the enlarged condyle, the neck of the left ramus, and a small portion of the posterior border of the coronoid process. At the site of section the bone was natural in size and consistence, but above that it became expanded into a broad oblong mass, with a rough flattened surface, measuring 1¾ inches from before back, and about 1 inch across. The outer surface of the mass, although nodular over the upper half, was smooth, and covered by a layer of dense bone, varying from 1 to 2 millimetres in thickness in front to a mere shell on the posterior border. The cancellous tissue filling the interior of the mass was in greatest quantity at the upper part, but the spaces were everywhere small, and the spicula of bone thick, so that the whole tissue was dense.

March 16th. The plug of lint was removed. There was a good deal of redness and swelling around the wound, extending to the cheek. The wound was syringed out and redressed.

The subsequent progress of the case was marked by increase of the swelling, and the formation of a slough at the bottom of the wound, which separated on March 27th, she was free from pain, and the sensation on the left side of the face was rather better; but she was not able to close the left eye completely. The swelling of the face gradually subsided, and the patlent nearly regained the power of closing the left eye. She was sent to the convalescent hospital at Eastbourne on April 12th.

Dr. Williams of Sherborne reported, some months later, that the movements of the jaw were very satisfactory, and that the patient had been able to take a situation, which her unsightly appearance had previously prevented.—*The British Med. Jour.*

## Figures and Tables

**Figure f1:**